# Expert ethos and the strength of networks: negotiations of credibility in mediated debate on COVID-19

**DOI:** 10.1093/heapro/daab095

**Published:** 2021-08-02

**Authors:** Jens E Kjeldsen, Øyvind Ihlen, Sine N Just, and Anders Olof Larsson

**Affiliations:** 1 University of Bergen, Bergen, Norway; 2 University of Oslo, Oslo, Norway; 3 Roskilde University, Roskilde, Denmark; 4 School of Communication, Leadership and Marketing, Kristiania University College, Oslo, Norway

**Keywords:** rhetoric, credibility, twitter, COVID 19, health authorities

## Abstract

For public health promotion to succeed, popular support is necessary and the chosen policies and measures have to be perceived as legitimate by the public. In other words, health authorities need to build on and sustain established trust when they recommend a certain policy. When the policy is criticized, this trust is challenged, and the authorities enter into a negotiation of credibility (ethos). In this article, we research a particular instance of such negotiation, drawing lessons for health promotion and for COVID-19 communication. We study a Norwegian television debate in which an MD presented harsh criticism of the health authorities’ chosen crisis response in the early phase of the pandemic. Unpacking the rhetorical constitution of the expert ethos of the MD and of the health authorities, respectively, we find that representatives of the authorities are more open to participation and better at connecting to everyday experiences than the MD, who primarily builds her expert ethos on mastery of scientific language and methods, combined with alarmist rhetoric. Further, we identify main tenets of the public’s reception of the debate through an analysis of 1961 tweets that commented on the program. The analysis indicates that public health authorities might maintain high levels of trust by rhetorically cultivating their positions within institutional *and* (social) media networks of expertise.

## INTRODUCTION

The importance of trust for the ability of a government to handle health crises can hardly be exaggerated, as has been emphasized in multiple studies of past crises (Deurenberg-Yap *et al.*, 2005; Mesch and Schwirian, 2015; Liu and Mehta, 2020). The centrality of trust in public institutions has been reconfirmed in the on-going COVID-19 pandemic, leading to calls for further studies of the interrelations of health promotion and trust (Van den Broucke, 2020). Closely connected to this issue is that of the credibility of the speaker (i.e. ethos), which becomes all-important when dealing with contingent matters where knowledge has to be established and opinions settled. When the subject matter is complex and contested, the twin issues of trust and credibility become dominant (Baumlin and Scisco, 2018). When different opinions on what is happening and what needs to be done are presented, citizens are not only persuaded by arguments tied to the issue, but also by the communicators’ credibility. The COVID-19 pandemic is such a contingent and uncertain matter. Accordingly, our research question is: *How is the ethos of health authorities and the public’s trust in these authorities negotiated when a chosen policy comes under attack?*

To answer this question, we have conducted a case study of a Norwegian debate program on the largest national television channel, the *NRK*, and the reception of this program by Twitter users. The program, *Debatten*, generally has between 300 and 500.000 viewers in a country of 5.3 million citizens (see https://m24.no/debatten-fredrik-solvang-nrk/513000-fulgte-fredrik-solvangs-koronasending-i-nrk-debatten-tirsdag–i-oyehoyde-med-folk/250088. See also: https://no.wikipedia.org/wiki/Debatten). On 17 March 2020, during the early phase of the COVID-19 pandemic, the *NRK* aired an episode of the program where an MD presented harsh criticism of the Norwegian public health authorities’ crisis response strategy. We investigate (i) how the authorities’ policy, and by implication the public trust in this policy, was challenged and defended in this program, and (ii) the reception of this criticism through Twitter users’ comments on the program, focusing on how the program affected the ethos of the authorities. Our analysis shows how the underlying basis of the Norwegian citizens’ high trust in the health authorities was activated to avert the criticism and sustain the credibility of the authorities. In this regard, the authorities’ use of rhetorical strategies to enhance their ethos of expertise and the support of this ethos in and through an established expert network are particularly important. Thereby, the MD’s attack on the authorities became positioned as an isolated instance, against which trust in the public health system, generally, and the chosen crisis response strategy, particularly, could be sustained.

The article is structured as follows: first, we introduce our theoretical lens, building on theories of trust, credibility and ethos; concepts of the rhetorical situation (Bitzer, 1968, 1980); and research of the ethos of expertise (Hartelius, 2011; Hartelius and Mitchell, 2014). Subsequently, we present our methodological approach, focusing on issues of data collection. The analysis begins with a brief description of the television program, establishing its rhetorical situation. Then we identify the involved actors’ main rhetorical strategies for establishing their ethos of expertise. Finally, we examine citizens’ reception of the program and their evaluation of the actors’ ethos in the Twitter debate that ensued. By way of conclusion, we point to general implications for how public authorities may defend their ethos and build and maintain public trust through appeals to expertise and expert networks.

## THEORY

### Trust, credibility and ethos

As a scholarly concept, trust has been defined as *acting with few precautions* [(Grimen, 2009), p. 19], and as *expectations that others will meet their commitments* (Hawley, 2014). The most well-known definition is probably Russel Hardins’ view of trust as *encapsulated interest*, which is grounded ‘in an assumption that the potentially trusted person has an interest in maintaining a relationship with the truster, an interest that gives the potentially trusted person an incentive to be trustworthy’ [(Hardin, 2006), p. 17]. Hence, trust is commonly seen as some form of expectation towards other people’s actions and their fulfilment of commitments. Such understandings generally see trust as interpersonal, delimited and situational, involving three parts: *someone trusts someone, in relation to something* [(Hardin, 2006), p. 19; (Grimen, 2009), p. 13f.]. In contrast to this, survey research on trust examines so-called generalized trust, measuring if people report trust in, for instance, ‘other people’, ‘the media’ or specific institutions, such as the health authorities. Hardin, among other scholars, have criticized such studies for missing the situational three-part-structure, thereby not being about trust in any real-life sense. Furthermore, Hardin criticizes survey research for being altogether atheoretical, leaving it to informants to use their own idiosyncratic sense of what trust is, when answering survey questions. He concludes that ‘there is relatively little to learn about trust’ from survey research on trust [(Hardin, 2006), p. 74].

However, survey studies do seem to demonstrate stability and correlation at the aggregate levels of generalized trust [(Nannestad, 2008), p. 418f.]. Norway, for instance, consistently comes out as a high-trust society both in surveys and experimental studies. Trust surveys, such as the ones conducted by the European Social Survey, consistently show how the Nordic countries are placed at the top (European Social Survey Round 9 Data, 2018). Norwegians give high scores in trust to both ‘other people’, and specific institutions. This is an important cultural circumstance for our study because we may assume a general high trust in the Norwegian health authorities. In fact, survey studies show that Norwegians had high levels of trust at the onset of the pandemic (see https://www.regjeringen.no/en/topics/koronavirus-covid-19/timeline-for-news-from-norwegian-ministries-about-the-coronavirus-disease-covid-19/id2692402/ and the data stems from an omnibus survey conducted by the firm *Respons Analyse* for the Norwegian Directorate of Health. The survey from March 30 had 739 respondents), in the spring of 2020.

Trust, then, as we approach it, is a relatively stable precondition, which forms the starting point and mental framework for any rhetorical communication. Thus, when we use the word trust, it denotes the expectation of the audience that health authorities in general have both the *skill* and *intention* to fulfil the commitment they have towards the citizens (Hawley, 2014).

Where, then, does trust come from? One source—the one we examine here—is rhetorical communication. More specifically, the public perceptions of health authorities’ *trustworthiness* and *credibility* (Chryssochoidis *et al.*, 2009; Renn, 2009) as they are attributed by audiences on the basis of the authorities’ communication. In the literature, these two dimensions are associated with a large number of more specific characteristics, including competence, expertise, knowledge, objectivity, fairness, consistency, sincerity, caring, empathy, compassion and goodwill (Renn and Levine, 1990; Covello, 2009).

In rhetorical theory, these traits are combined in the concept of *ethos*. Ethos is the communicator’s rhetorical establishment of credibility, through demonstration of *competence*, *character* and *goodwill* (McCroskey and Young, 1981; McCroskey and Teven, 1999; Aristotle, 2007). Ethos, then, may be viewed as the specific dimensions of attributed credibility resulting from rhetorical communication. Such communication must necessarily build on some form of initial trust; however, the communication itself may also establish or further develop trust. Thus, trust can be perceived as both the starting point for and the outcome of rhetorical communication (Hoff-Clausen, 2013). Similarly, ethos refers to both the communicator’s attempt to build credibility and the attitude that the audience holds towards the communicator at any point in time. This means that ethos is not a fixed quality in a sender or a text but is constantly negotiated (Delia, 1976), with trust being one central ingredient in and outcome of this negotiation.

### The theory of the rhetorical situation

Risk communication researchers agree that institutional trust is a key component for citizens’ reception of public authorities’ risk response (Deurenberg-Yap *et al.*, 2005; Mesch and Schwirian, 2015; Liu and Mehta, 2020). Still, the particulars of how institutional communicators can draw on existing public trust and/or build trust through their communication remain underdeveloped. To facilitate such development, there is a need for more research on *the rhetorical situation* of public authorities’ risk communication, as this can shed light on the purposes of this communication and the condition that enable and delimit its success.

The rhetorical situation, as a specific theoretical concept, has three constitutive elements (Bitzer, 1968): First, there is a pressing problem, an *exigence*, which is ‘an imperfection marked by urgency’ that ‘demands’ a rhetorical response. Crucially, for the problem to be rhetorical, it has to be solvable (wholly or partially) with the help of rhetoric. An exigence consists of two elements: a factual condition and a relation to some interest [(Bitzer, 1980), p. 28]. This means that different possible rhetors may see different exigencies, and thus interpret the whole situation differently. In the context of COVID-19, the number of infected people would be a factual condition that establishes an exigence while evaluation of the condition and advocacy of the preference of action makes the situation rhetorical as the rhetor may choose to relate the facts to various different interests in any number of ways.

The second element is the *audience*, defined as the individual(s)/group(s) that the rhetor wishes to persuade or influence to think differently and/or take action. As a minimum, the audience has to share the rhetor’s belief that something is a problem and needs to be addressed. In Bitzer’s definition, the audience is further narrowed to those who are, indeed, able to solve the problem and who can be persuaded by the rhetor. This latter element indicates that the audience must always hold a modicum of trust in the rhetor, which implies that rhetorical efforts can lead the audience to give or take away trust from the rhetor. Trust, then, is both a prerequisite for and an effect of persuasion (Hoff-Clausen, 2013).

The third element is *constraints*, which are the mental, physical, and cultural contingencies that the rhetor needs to relate to when addressing the exigence. The rhetor’s central task is to ‘discover and make use of proper constraints in his message in order that his response, in conjunction with other constraints operative in the situation, will influence the audience’ [(Bitzer, 1980), p. 23f.]. Rhetorically, then, constraints function as opportunities and limitations for the rhetor. Taken together, the three elements—problem, audience and constraints—have been said to prescribe certain responses that can ‘fit’ (i.e. become persuasive in) the situation (Bitzer, 1968, 1980).

### The ethos of expertise

As mentioned, the ethos of the rhetor is particularly important in complex situations with high uncertainty. When policy recommendations are (partially) based on scientific knowledge, but disagreements exist within the relevant scientific communities, as is the case with the COVID-19 pandemic, establishing an *ethos of expertise* is particularly salient. To study this aspect, we apply Hartelius’ [(Hartelius, 2011), p. 18ff.] six congruities that describe what experts *do* rhetorically. Here, congruities are defined as common patterns in experts’ rhetorical repertoire, or ‘cross-contextual similarities in experts’ discursive means of constructing expertise’ [(Hartelius and Mitchell), 2014, p. 297]. We use the congruities to examine the constitution of expertise in general, independently of who does the constituting, seeing them as discursive techniques for establishing ethos of expertise.

The first congruity is *expert network*s. Expertise is constituted by ‘associating oneself strategically with other experts as well as with other areas of expertise’ (p. 18). The second is *expert techne*. This signifies establishing expertise by explicating ‘epistemologies and methodologies’ belonging to one’s field of expertise (p. 19). To rhetorically establish their expertise, experts ‘state what they know, how they know it, and how they practice or implement what they know’ (p. 20). Third, *expert pedagogy* means that experts not only share epistemology and methodology, but also share ‘*how* they know what they know’ (p. 23). An open sharing of process and the uncertainties of method and knowledge may reinforce the sense of expertise. The fourth congruity, *deference/participation*, signifies the choice of experts to either invite the audience to acquiesce or to get involved. Since expertise and professional knowledge is by nature specialized, complex and difficult for the nonprofessional to understand, deference is the most common strategy. However, in some instances experts will encourage an audience to participate. Such participation, of course, will require *expert pedagogy* and explanation of *expert techne*. The fifth congruity is *expertise as fitting response*. As we know from Bitzer, a rhetorical situation has a defect or obstacle, something waiting to be done, and this ‘imperfection’ can be addressed by rhetorical communication. In the constitution of expertise, experts ‘identify or construct a rhetorical situation in which their expertise is the most fitting response’ [(Hartelius, 2011), p. 23]. Finally, expertise is constituted by creating *relevance to everyday life*. Experts, Hartelius explains, must orient themselves and their subject matter in ways that make them recognizable and relatable (p. 27): ‘The more relevant an expert seems to the public, the more powerful she will be’ (p. 29).

In the following, we will use the rhetorical situation as a general framework within which we explore how the six congruities function in the constitution of the ethos of expertise by the participants in the program as well as through the program’s framing of them and how this is reflected or not in the tweets commenting on the program.

## METHODOLOGY

Our empirical approach is that of a case study, focusing on a television program and the comments that followed on Twitter in the first three days after the debate (The program, with Norwegian subtitles, is available on https://tv.nrk.no/serie/debatten/202003/NNFA51031720. All quotes are translated from Norwegian to English by the authors.). The case itself is of interest, we argue, because it stems from a period in the COVID-19 pandemic when the World’s nations had to decide on which policy to pursue. The virus and the public response to it were surrounded with uncertainty and disagreement between both health professionals and responsible politicians. Our case focuses on how one MD criticized the public response chosen by the Norwegian health authorities, thereby challenging their ethos and, potentially, decreasing public trust in the authorities, while simultaneously seeking to strengthen her own credibility. Our study is a version of what has been called *rhetorical reception analysis*, which aims to connect textual rhetorical analysis with analyses of different types of reception analysis (Kjeldsen, 2016, 2018b). The aim is not to establish causal effect, but to explore connections between rhetorical utterances and their reception. In our case, we explore the connections between the rhetorical ethos-work in the program and the reception as it manifested itself in the ensuing twitter-debate [(cf. Kjeldsen, 2018a), p. 29].

We conducted a rhetorical analysis of the television program using the theoretical apparatus presented above: first, by determining the rhetorical situation, then by examining the program through the lens of the congruities. We then gathered tweets commenting on the program by searching for hashtags related to the program—#NRKdebatten and #NRKdebatt—from 17–20 March. Data collection was done by means of the rTweet package for the R programming language (https://rtweet.info), and was undertaken on March @20 so as to overcome the temporal limitations of the Twitter Application Programming Interface [e.g. (Venturini *et al.*, 2018)]. The data gathering process resulted in 1961 tweets carrying at least one of the two mentioned hashtags. These tweets were then sorted according to their popularity—understood here as the number of times that they had been marked as ‘favourite’ and/or the number of times they had been redistributed by other users, referred to as retweeted.

With regards to Twitter, and especially the use of thematic keywords like hashtags on the specified platform, it is important to remember that such practices cannot be considered as common among Norwegian citizens. Indeed, studies have shown that Twitter use is an elite practice—especially in relation to issues like the one discussed here (Larsson & Moe, 2014). In our case, however, Twitter is relevant, since we are examining expert ethos and the strength of networks.

A research assistant and the lead author of this article carried out a preliminary inductive coding by reading through the gathered tweets. We established tentative categories, tested these against the full material and then revised the categories. This way of moving inductively and deductively between the material and possible categories, allowed us to establish the final nine categories, involving negative/positive sentiment towards the MD, the health authorities and the public broadcaster as well as feelings of uncertainty and anxiety. The categories are: (i) No sentiment/other, (ii) Positive towards NRK/reporter, (iii) Negative towards NRK/reporter, (iv) Positive towards MD, (v) Negative towards MD, (vi) Positive towards health authorities, (vii) Negative towards health authorities, (viii) General uncertainty of facts and (ix) Anxiety/panic.

As we demonstrate below, a broad portion of the sampled tweets fall into the first category ‘No sentiment/other’. Although this could appear to threaten the validity of the study, since such a catch-all-category may miss out on data that is pertinent to the study, this is not the case here. First, because we are searching specifically for sentiments in relation to relevant actors. Secondly, because our categories for these are also broadly encompassing. Furthermore, the main objective of the coding and categories was to get to the different types of rhetoric in the tweets. An intercoder reliability test was carried out by a third research assistant who re-coded 10% of the tweets. This resulted in a weighted Kappa intercoder agreement of 0.547 (‘moderate agreement’), which is considered to be sufficient (Gwet, 2014).

The data collection was carried out in compliance with the guidelines for research ethics concerning collection and treatment of data as set out by the Norwegian National Ethical Committees for the Social Sciences and Humanities.

## ANALYSIS

In the following analysis, we first explain the rhetorical situation. Then we examine the MD’s critique of the authorities and their policies, explaining how her expertise is constituted, and how the expertise of the authorities is challenged. We move on to showing how the authorities rhetorically attempt to reconstitute their ethos and retain trust in the proposed policy. Finally, we examine the Twitter reactions to this negotiation.

### The rhetorical situation

On 26 February 2020, the first case of COVID-19 was registered in Norway (https://www.regjeringen.no/en/topics/koronavirus-covid-19/timeline-for-news-from-norwegian-ministries-about-the-coronavirus-disease-covid-19/id2692402/). On 12 March 2020, as the situation escalated, the Government introduced what it called the most comprehensive measures ever in peacetime. Schools and kindergartens were closed down, sports and cultural events were called off, bars and hairdressers were forced to close, and so forth. Still, some critics were asking for even stronger measures, thus questioning how the health authorities handled the pandemic.

In Norway, the health authorities include the Norwegian Institute of Public Health (NIPH) and the Norwegian Directorate of Health (NDH), which both are agencies under the Ministry of Health and Care Service. NIPH is a national competence institution that also carries out research, whereas NDH is an executive agency and professional authority.

The national sentiment towards the health authorities is reflected in a survey from the Norwegian Directorate of Health, indicating high levels of trust at the onset of the pandemic (in February 92% had full, high or some trust in the authorities). On 7 March, the percentage of respondents that expressed full trust had fallen from 38% to 16%. Still, 83% had full, high or some trust (The data stems from an omnibus survey conducted by the firm *Respons Analyse* for the Norwegian Directorate of Health. The survey from March 30 had 739 respondents.).

In a study from the *Norwegian Citizen Panel* (NCP) carried out 20 March (The Survey began on 20 March and was ended on 29 March. It involved valid 12 051 respondents. On *NCP* see: https://www.uib.no/en/citizen.), informants were asked how much trust they had in whether ‘the health authorities (NHIP and NDH) handles [the spread of the Coronavirus] in a good way’. 22.7% said ‘very high trust’, 57.0 said high trust, 16.4% said ‘some trust’, while only 2.6% said ‘low trust’ and 0.4% had ‘no trust’. In the same survey, 63% had ‘very high’ or ‘high’ trust in the government’s handling of the crisis, and 25% had ‘some trust’.

In sum, the rhetorical situation of the early Norwegian response to the pandemic was characterized by general agreement on the exigence and its solution: The factual condition was the spread of COVID-19 as determined by the health authorities. The related interest, seen from a national perspective, was a wish to help the country and its citizens through compliance with the policy recommended by government and health authorities.

### The critique of authorities and policy, and the establishment of the expertise of the MD

On 17 March 2020, the credibility of the authorities was challenged. The program *Debatten* on *NRK* was announced with a title suggesting that the health authorities had chosen the wrong strategy: ‘Corona—are the measures strict enough?’ From the outset, the program signals a discrepancy between Norwegian health authorities and international *expert networks*. To illuminate the issue, the *NRK* had invited the hitherto publicly unknown medical doctor Gunnhild Alvik Nyborg (hereafter: the MD) to evaluate the public response to the COVID-19 pandemic. The MD is the only person in the studio, and by way of introduction the host turns to her and says: ‘First, I think that you should be allowed to explain why you have a background that means that we and the authorities should listen to you’. The MD replies:

I have a quite broad background; I have worked with related matters from a number of different perspectives. I have worked as an MD in several parts of the country. I have a doctorate in pharmacoepidemiology. Now I work as a researcher with T-cells, which are those cells that concern virus replication. And I also have a university degree in economics. So, I have looked at this from several different perspectives and have become very concerned.

Because the MD is introduced immediately after the discursively established discrepancy between the Norwegian crisis response and the recommendations of the international expert network, she is rhetorically connected to the international experts that Norwegian authorities are allegedly not listening to. At the same time, her answer associates her strategically with what are supposedly relevant areas of expertise. Here, the doctorate and her current work as a researcher are vital prerequisites of *expert techne*. Importantly, she is not only a researcher, but also has first-hand experience as an MD, thus creating something of a *relevance to everyday life*.

The program proceeds with a segment of 32 min that consists of an interview with the MD. Here, she redefines the rhetorical situation by disputing the significance of the facts of the rhetorical exigence: she accepts the numbers and the factual account of the present situation as described by the health authorities; however, she accuses the authorities of not fully grasping the severity of the situation and, accordingly, not taking sufficient measures. The rhetorical problem, from her point of view, is to convince the audience that the current measures are insufficient. In order to succeed, the MD not only needs to put forward a compelling case (logos), but also to make the audience realize and feel the seriousness of the situation (pathos), and especially to establish her credibility (ethos). She must demonstrate the expertise needed to back up her claims.

The MD establishes the seriousness of the situation by saying that 150.000 people may die in Norway, stating that ‘this is war’, and using metaphors recalling the Norwegian experience from the Second World War by alluding to the situation on the morning of the invasion when the German cruiser Blücher sailed into the Oslo Fjord and met armed resistance. Not acting, she claims, would be like letting Blücher pass without firing a single shot. Thus, appeals to emotion (pathos) support the MD’s ethos appeals to establish the credibility of her view of the situation.

Simultaneously, the questions of the reporter and the explanations and arguments by the MD establish her ethos through *expert techne* and *expert pedagogy.* Here, expert techne is performed in a rare instance of complex information in a debate program: The MD is invited to interpret and explain complicated equations projected on a screen. Her explanation of the numbers and equations that are important for our understanding of matters such as infection rate and spread of virus amounts to an elaborate demonstration of expert techne. As the explanation is somewhat difficult to follow, the MD does not make full use of the possibility to demonstrate expert pedagogy.

The MD’s criticism is supported by *NRK*’s framing of the interview. As we saw, the introduction in the program directly questioned the strategy of the authorities and let the MD explain her expertise. The enabling approach from the *NRK* is also clear from the character of the questions, which are mostly of the type known in conversation analysis as *softball questions* that advance adversarial viewpoints ‘at most mildly or half-heartedly and in a way that eases response’ [(Clayman and Fox, 2017), p. 20].

The attack on the health authorities by the MD, then, is supported by the journalistic framing. Thus, the MD and the journalist work together to establish her as a credible and trustworthy figure on the matter of the COVID-strategy. Her criticism—supported in the establishment of her expert ethos—is the fitting response to the rhetorical situation as she redefines it.

### The authorities’ reconstitution of expertise and proposed policy

The next segment of the program lasts 27 min. Although the MD is still present in the studio, representatives from NIPH and NDH are brought in via video link. From their point of view, the rhetorical problem in the specific situation created by the program is the critique of their policies and undermining of their ethos. The fitting response, for them, is to refute the attack, retain trustworthiness and reassure the audience that adequate measures have been taken to mitigate the pandemic. The question, then, is how to do this in the most credible and persuasive way?

The public authorities are placed in a position of defence by their chronological appearance in the program. After we have heard the MD’s attack, they are given the opportunity to respond. This negative framing is enhanced through *hardball questions* from the host. According to conversation analysis such questions ‘advance an adversarial viewpoint vigorously in a way that is ostensibly difficult to counter’ [(Clayman and Fox, 2017), p. 20]. In facing the program’s negative framing of the policy and the ethos of the authorities, the two representatives maintain their calm. They answer all questions in an open and agreeable manner, making minor concessions that enhance their appearance of reasonableness while standing firm on main issues and insisting on the appropriateness of the policies.

These rhetorical moves are present from the outset of this segment, when the word is given to the NIPH-representative. He partially accepts the MD’s critique, agreeing that ‘we do not have the full overview over who is infected’. He also explicitly agrees that all the premises (assumptions) on which the chosen policy is built can be discussed. However, he points to a ‘serious flaw’ in the argument of the MD, namely that she says that the number of cases in Oslo (the capital of Norway) in particular is increasing tremendously. The increase, he argues, is due to imported cases, not an indication that people are getting infected in Oslo.

After the first comments of the two representatives, the MD says that she is ‘not at all reassured by this. We do not test’. The program host seems to take for granted that the MD is right and directs an adversarial question to the NDH Director General (DG):

And how long then, can you insist that … you say that you have tested about 20.000. Everybody can see that 20.000 is very little when you estimate that 2.2 million will get the disease.

The DG answers that ‘We increase the test capacity all the time’. The host again responds in an adversarial way: ‘But you have said that for weeks?’ And follows up the DG’s response with an invitation to the MD to have the last say in the matter.

In general, the representatives of NIPH and NDH do not do much explicit rhetorical work in demonstrating *expert techne* and *expert pedagogy*. At this point in time, however, they had already been established as two of the health authorities’ main spokespersons, and we may assume that these two congruities are taken for granted. Both representatives, however, support their expert ethos by enhancing the *relevance to everyday life* of the proposed policy, especially through the use of plain language and a calm tone of voice, which is notably different from the rather alarmist language and tone pathos used by the MD.

The language and tone of the participants also affect the constitution of *deference and involvement*. None of the parties explicitly asks the audience to defer (to acquiesce) nor are there invitations to get involved (to participate). The determination and certainty of the MD and the seriousness of her rhetoric; however, implicitly requires *deference*. The situation is presented as so serious and the problem so complex (as illustrated with the equations) that the authorities and citizens (i.e. the audience) are constituted as having to defer to her position. The representatives of NIPH and NDH, conversely, give concessions and talk about dilemmas, establishing a more open expert role that offers the audience the opportunity to make up their own minds.

Even though the MD—with help from *NRK—*seeks to establish herself a part of an *expert network* (as described earlier), she nonetheless comes across as a single voice. The public did not know her before the program, and she is not directly connected with experts who actually work with the problem. She merely references what other experts say. This makes her appear as a kind of outsider-expert, a participant without actual connections to the most relevant expert networks. In contrast to this, the image of the representatives of NHIP and NHD—seen beside each other on the screen, which is literally hanging above the host and the MD—becomes a visual confirmation that they belong to the same national network of experts; a network, which the population is already familiar with. The GD of NHD expresses the functionality of this network, when he says (43:40): ‘of course our elected representatives also have to take part of the responsibility of the decisions we will make in the future’. Thereby, he reminds the public that he and the health authorities are closely connected to political leadership. Furthermore, they are connected to experts nationally and internationally:

What we have to do is to listen to the best experts we have, who also collaborate with very competent experts other places in the world. And on this basis make our decision.

This establishes the health authorities as part of a strong national and international expert network, meaning the population can trust that the authorities have the necessary knowledge and competence to actively and appropriately deal with the situation at hand.

In sum, the representatives for the health authorities answer the attack from the MD by building their ethos of expertise in ways that are quite different from the construction of hers. Although similarities exist in the appeals to expert techne and expert networks, they counter her alarmist tone and call for more radical measures with openness towards the voiced concerns coupled with calm reassurance that they are already doing all that is possible—and that this is enough. As we will now show, these differences in particular strategies affect how their claims to expertise is received and negotiated on Twitter.

### The Twitter reception

As indicated, the program caused a stir. On the one hand, some actors sought to distance themselves from the MD; most notably, her employer went public to state that she was not speaking as their representative (NTB, 2020a,b). On the other hand, traditional media reported that she was receiving support from other health professionals (Kvale, 2020). Another strand of the debate focused on the format of the program. For instance, the representative from NIPH complained, on Twitter, that the program had not been balanced. Incidentally, this tweet is the second-most retweeted message in our material. The *NRK* agreed that the program had not been ideal in this sense, but stood firm on its overall relevance and appropriateness (NTB, 2020a,b).

**Fig. 1.**
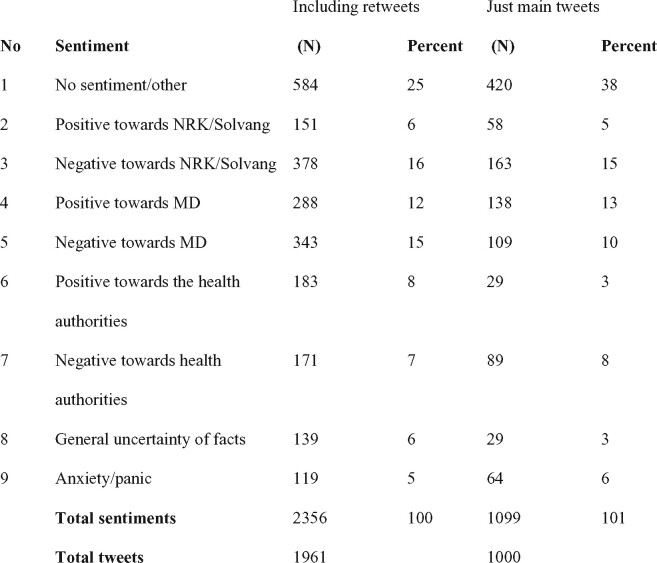
Expert ethos and the strength of networks: negotiations of credibility in mediated debate on COVID-19.

Focusing on the debate as it unfolded on Twitter, we coded the material according to the expressed sentiments. The distribution can be seen in Figure 1.

Most of the tweets did not express any sentiment towards the actors we examine (38% of the main tweets and 25% of the retweets). This indicates that the debate on twitter was not primarily oriented towards the actors and their ethos but directed at other issues. When it comes to sentiments directed at the actors, the most prominent sentiments expressed are negative towards the *NRK* (15%, and including retweets: 16%) followed by negative sentiments towards the MD (10%, and including retweets: 15%). Further, these two sentiments are most frequently combined in the tweets (82 instances, incl. retweets). Thus, tweets that are critical of the MD tend to be critical of *NRK* as well. The second most common combination of sentiments in the same tweet is negative to *NRK*, negative to MD, and positive to health authorities (68 instances). Thus, negative evaluations of the MF and the *NRK* are not only correlated with each other, but connected to positive evaluation of the health authorities as well. Many of the Tweets criticize the *NRK* for using a sensationalist approach, combining this critique with distrust in the MD. This, we argue, is particularly interesting since the program had a bias against the policy and ethos of the authorities and in favour of the arguments of the MD*.* In the tweets, however, negativity towards the authorities was very low (8%, and including retweets: 7%).

**Fig. 2.**
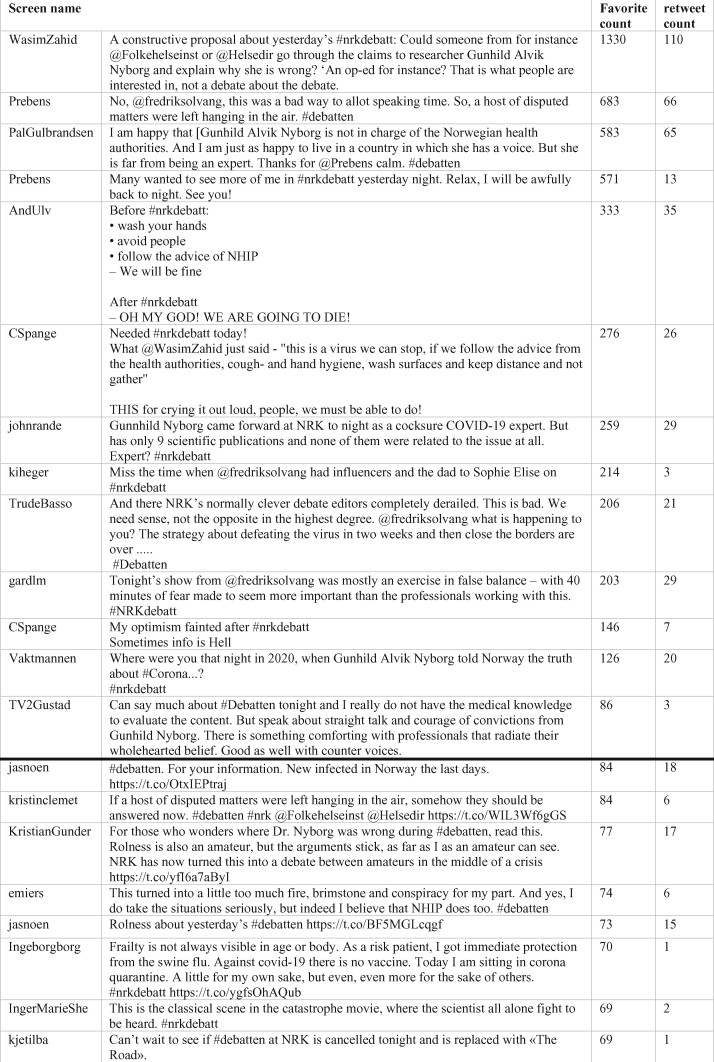
Expert ethos and the strength of networks: negotiations of credibility in mediated debate on COVID-19.

It is also interesting that in the most favourited tweets, the expertise of the MD was questioned and the credibility of the authorities was emphasized explicitly (see Figure 2).

This speaks directly to the first two parts of our analysis, showing how the Twitter-public is more favourable towards the authorities’ strategies than those of the MD (as supported by the *NRK*). Thus, even if the program seemed to favour the MD, building her ethos at the expense of the authorities’, the Twitter response reversed the dynamic.

The impression from Figure 1 is strengthened when studying the content of the 20 Tweets that have the highest favourite count, and typically are the most retweeted as well (see Figure 2). The tweet that tops the list urges the public health authorities to rebut the claims made by the MD. This tweet has been favourited and retweeted nearly twice as many times as the second most popular tweet (1330 favourites and 110 retweets, versus 683 favourites and 66 retweets). Other top tweets challenged the expert status of the MD; one labelled her an ‘amateur’, whereas another pointed out that the MD ‘has only nine scientific publications and none of them were related to the issue at all’. Some Twitter users did show support for the MD, e.g. finding that ‘There is something comforting with professionals that radiate their whole hearted belief’. This, however, remained a minority position with the emerging consensus being well-captured by another popular tweet: ‘I am happy that [the MD] is not in charge of the Norwegian health authorities. And I am just as happy to live in a country in which she has a voice’ (see Figure 2).

## DISCUSSION AND CONCLUSION

In moving from the particular analytical findings to consideration of their general implications, we emphasize that we are not claiming a causal effect based on this single program. Rather, it is likely that the high levels of institutional trust in the Norwegian health authorities serve as a ‘cushion’ for the authorities that mitigate specific instances of critique. We emphasize this point to reinforce the theoretical position that trust and ethos go hand in hand. However, the higher initial trust in known representatives of public authorities than in one particular and unknown MD is not the only reason that the authorities did not suffer from this particular attack.

This study provides an important illustration of some factors involved in negotiations of trust and ethos in a particular situation in a media landscape where social media plays a crucial role. Our study points to several issues of relevance for health authorities having their policy and ethos questioned. First, it indicates that all six congruities can and should be used; however which, when and how depend on the rhetorical situation and the character of the exigence. Second, it points to ways in which expert ethos may be operationalized. Although we do not claim quantitative generalizability, our study supports existing theory and suggests the following rhetorical rules of thumb: (i) Use open, invitational rhetoric, (ii) Use plain language to anchor expertise in everyday contexts and (iii) Create allies and networks. The two first points follow directly from our analysis of the program in which we found these two strategies to be the main differences between the ethos appeals of the MD and the representatives of the public authorities’ expressions of their expert ethos.

The third aspect merits further attention, as it points towards a possible reconceptualization of the ethos of expertise that emphasizes expert networks, but also reconsiders the character and rhetorical value of expert networks. Hence, the specific analytical point is that the Norwegian health authorities were not only able to draw on an institutional expert network, using the same references to international experts as the MD, but also connected their expertise to the network of Norwegian political incumbents. Thereby, their position in the network is supported by political authority as well as scientific expertise. Further, and as demonstrated in the analysis of the Twitter reception, the public health authorities are centrally positioned within the network of Twitter users. Although this is not an expert network in the classical sense, it nonetheless functions to bolster the authorities’ ethos of expertise in the particular case. In fact, the Twitter users’ support of the authorities seems to outweigh the classical mass media’s (i.e. the program’s) support of the MD. Thus, we argue, finding new (social) media allies to publicly promote health authorities’ ethos may be key to ensuring public support for proposed policies and measures in the current crisis and beyond. 
